# *Nrf2*-Knockout Protects from Intestinal Injuries in C57BL/6J Mice Following Abdominal Irradiation with γ Rays

**DOI:** 10.3390/ijms18081656

**Published:** 2017-07-31

**Authors:** Wenyan Yang, Zhijuan Sun, Bing Yang, Qin Wang

**Affiliations:** 1 Tianjin Key Laboratory of Radiation Medicine and Molecular Nuclear Medicine, Institute of Radiation Medicine, Chinese Academy of Medical Sciences and Peking Union Medical College, Tianjin 300192, China; ywy920913@163.com (W.Y.); sunzj@irm-cams.ac.cn (Z.S.); 2 Department of Cellular Biology, School of Basic Medical Sciences, Tianjin Medical University, Tianjin 300070, China; yangbingtj@aol.com

**Keywords:** Nrf2, intestinal injury, abdominal irradiation, intestinal stem cells, NF-κB

## Abstract

Radiation-induced intestinal injuries (RIII) commonly occur in patients who suffer from pelvic or abdominal cancer. Nuclear factor-erythroid 2-related factor 2 (Nrf2) is a key transcriptional regulator of antioxidant, and the radioprotective role of Nrf2 is found in bone marrow, lung, and intestine, etc. Here, we investigated the effect of *Nrf2* knockout on radiation-induced intestinal injuries using *Nrf2* knockout (*Nrf2^−/−^*) mice and wild-type (*Nrf2^+/+^*) C57BL/6J mice following 13 Gy abdominal irradiation (ABI). It was found that *Nrf2* knockout promoted the survival of irradiated mice, protected the crypt-villus structure of the small intestine, and elevated peripheral blood lymphocyte count and thymus coefficients. The DNA damage of peripheral blood lymphocytes and the apoptosis of intestinal epithelial cells (IECs) of irradiated *Nrf2^−/−^* mice were decreased. Furthermore, compared with that of *Nrf2^+/+^* mice, *Nrf2* knockout increased the number of *Lgr5^+^* intestinal stem cells (ISCs) and their daughter cells including Ki67^+^ transient amplifying cells, Villin^+^ enterocytes, and lysozyme^+^ Paneth cells. Nuclear factor-κB (NF-κB) was accumulated in the crypt base nuclei of the small intestine, and the mRNA expression of NF-κB target genes *Bcl-2*, *uPA*, and *Xiap* of the small intestine from irradiated *Nrf2^−/−^* mice were increased. Collectively, *Nrf2* knockout has the protective effect on small intestine damage following abdominal irradiation by prompting the proliferation and differentiation of *Lgr5^+^* intestinal stem cells and activation of NF-κB.

## 1. Introduction

The small intestine is one of the most sensitive organs of the body to ionizing radiation (IR). The radiotherapy for abdominal or pelvic cavity tumors may destroy intestinal absorption and barrier function and cause radiation-induced intestinal injuries (RIII) [1]. The major symptoms of radiation-induced intestinal injuries include diarrhea, nausea, bleeding, abdominal or rectal pain, and electrolyte loss, etc. These adverse side effects of radiotherapy limit the effective radiation dosage applied to eradicate tumors. Therefore, radiation-induced intestinal injuries seriously interfere with the treatment of patients with abdominal or pelvic cavity tumors and reduce the patients’ quality of life.

Nuclear factor-erythroid 2-related factor 2 (Nrf2), the redox sensitive transcriptional activating factor, is a member of the cap “n” collar (CnC) family that is composed of the transcription factors SKN-1, NRF1, NRF2, NFE2, NRF3, CncC, BACH1, and BACH2. These factors are characterized by a leucine zipper protein-protein dimerization domain, as well as CnC and basic domains that confer DNA binding activity [2]. Nrf2 heterodimerization with small musculoaponeurotic fibrosarcoma protein (sMaf) binds to *cis* antioxidant response elements (AREs) to govern the expression of Nrf2 target genes [3], which are involved in a wide range of antioxidant response and xenobiotic metabolism [4].

It is generally accepted that exposure to IR can produce radical oxygen species (ROS), including hydroxyl radicals, hydrogen peroxide and superoxide, which contribute to cell and tissue injury. Hydroxyl radicals can result in single strand breaks, double strand breaks, protein DNA crosslinks, as well as other types of DNA damage. These DNA damages caused by hydroxyl radicals, and hydrogen peroxide and superoxide as a secondary consequence of IR, can induce cell apoptosis [5]. Under oxidative stress, Nrf2 upregulates the antioxidant enzymes and detoxifying proteins. These proteins can scavenge radical oxygen species to reduce DNA damage and cell apoptosis following IR [6]. Therefore, Nrf2 has a radioprotective effect on cell and tissue damage induced by IR.

It was reported that the activation of Nrf2 mitigated radiation-induced lung injury [7,8], hematopoietic system injury [9,10,11], and bone loss [12]. Some studies showed that Nrf2 activation alleviated radiation-induced intestinal damage in mice post total body irradiation (TBI) [13,14,15,16]. Conversely, Berhane et al., reported that *Nrf2* knockout increased the radioresistance of bone marrow stromal and hematopoietic progenitor cell lines derived from *Nrf2^−/−^* homozygous deletion recombinant-negative mice [17]. Until now, the role of Nrf2 on radiation-induced intestinal injuries remains poorly understood. Here, we explored the effect of Nrf2 on intestinal injuries to elucidate whether Nrf2 plays a radioprotective role in radiation-induced intestinal injuries. In this study, a mouse model of radiation-induced intestinal injuries in *Nrf2* knockout (*Nrf2^−/−^*) mice and wild-type (*Nrf2^+/+^*) C57BL/6J mice was built by exposure to 13 Gy abdominal irradiation (ABI). The 30-day survival rate of mice, the crypt-villus structure of the small intestine, the apoptosis of intestinal epithelial cells (IECs), and the proliferation and differentiation of *Lgr5^+^* intestinal stem cells (ISCs) of the small intestine were respectively assessed.

## 2. Results

### 2.1. Nrf2 Knockout Improved the Survival of Mice after Abdominal Irradiation

**Figure 1:**
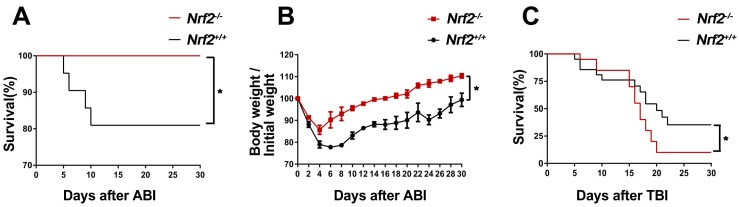
The effect of *Nrf2* knockout on the survival rates of mice after exposure to abdominal irradiation or total body irradiation. (**A**) Kaplan–Meier survival curve of *Nrf2^+/+^* mice and *Nrf2^−/−^* mice after 13 Gy abdominal irradiation. Mice (*n* = 21 mice/group) were treated with 13 Gy abdominal irradiation and monitored continuously for 30 days to determine survival rates. The data were expressed as a percentage of surviving mice; (**B**) mice (*n* = 21 mice/group) were treated with 13 Gy abdominal irradiation and monitored continuously for 30 days to record the body weight of survival mice. The data were expressed as body weight/initial weight; and (**C**) the Kaplan–Meier survival curve of *Nrf2^+/+^* mice and *Nrf2^−/−^* mice after 7.2 Gy total body irradiation. Mice (*n* = 21 mice/group) were treated with 7.2 Gy total body irradiation and monitored continuously for 30 day to determine survival rates. The data were expressed as the percentage of surviving mice. * *p* < 0.05, *Nrf2^−/−^* group versus *Nrf2^+/+^* group.

To investigate whether Nrf2 has radioprotective effect in vivo, we first analysed the 30-day survival of *Nrf2^−/−^* mice and *Nrf2^+/+^* mice following 13 Gy abdominal irradiation. As shown in Figure 1A, all *Nrf2^−/−^* mice survived after exposure to abdominal irradiation. In contrast, 80% of *Nrf2^+/+^* mice survived 10–30 days after abdominal irradiation, which was significantly lower than that of the irradiated *Nrf2^−/−^* mice (*p* < 0.05). At the same time, the body weight of survival mice was monitored for 30 days after abdominal irradiation. The body weight of survival mice was decreased four days after abdominal irradiation, subsequently, the body weight gradually increased in the following days. The speed of body weight recovery in *Nrf2^−/−^* mice was significantly faster than that in *Nrf2^+/+^* mice (*p* < 0.05) (Figure 1B). These results suggested that *Nrf2* knockout improved the survival of mice treated with abdominal irradiation and their body weight recovered rapidly.

In addition, the 30-day survival of *Nrf2^−/−^* mice and *Nrf2^+/+^* mice exposed to 7.2 Gy total body irradiation was observed. As shown in Figure 1C, the survival of *Nrf2^−/−^* mice was higher than that of *Nrf2^+/+^* mice within 15 days following 7.2 Gy total body irradiation. However, 7.2 Gy total body irradiation resulted in 14% survival in *Nrf2^−/−^* mice by day 30. In contrast, the survival rate of *Nrf2^+/+^* mice at day 30 was 43%, which was significantly higher than that of the *Nrf2^−/−^* mice (*p* < 0.05). The data suggested that the effect of abdominal irradiation and total body irradiation on the survival of *Nrf2^−/−^* mice were totally different, that was, *Nrf2* knockout protected from intestinal injuries induced by abdominal irradiation, but did not protect from tissue damage induced by total body irradiation.

### 2.2. Nrf2 Knockout Increased the Immune Organ Coefficient of Mice after Abdominal Irradiation

**Figure 2:**
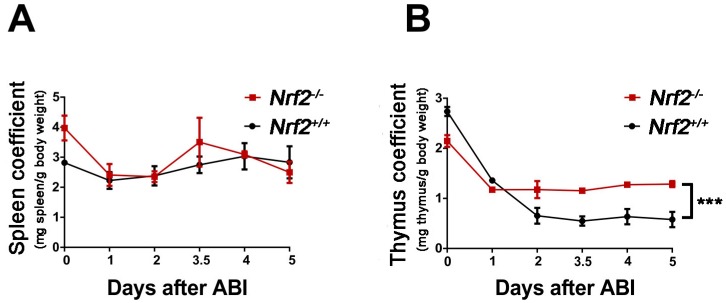
The effect of *Nrf2* knockout on the immune organ coefficients of mice after exposure to abdominal irradiation. (**A**) The spleens were collected and weighted after *Nrf2^+/+^* mice and *Nrf2^−/−^* mice were euthanized 1, 2, 3.5, 4, and 5 days post-abdominal irradiation. The spleen coefficient was calculated by spleen weight (mg)/mice weight (g). The data were expressed as mean ± SD (*n* = 4 mice/group); (**B**) the thymus were collected and weighted after *Nrf2^+/+^* mice and *Nrf2^−/−^* mice were euthanized 1, 2, 3.5, 4, and 5 days post-abdominal irradiation. The thymus coefficient was calculated by thymus weight (mg)/mice weight (g). The data were expressed as mean ± SD (*n* = 4 mice/group). *** *p* < 0.001, *Nrf2^−/−^* group versus *Nrf2^+/+^* group.

To investigate the immune function of *Nrf2^−/−^* mice and *Nrf2^+/+^* mice post-abdominal irradiation, the spleen coefficient and thymus coefficient of mice at 1, 2, 3.5, 4 and 5 days after abdominal irradiation were calculated. The spleen coefficient decreased rapidly from the irradiated mice one day after exposure to abdominal irradiation, subsequently, the coefficient gradually increased in the following days. However, there was no distinct difference in the spleen coefficient of *Nrf2^−/−^* mice and *Nrf2^+/+^* mice (Figure 2A). While the thymus coefficient of *Nrf2^+/+^* mice was decreased rapidly two days after abdominal irradiation, the value remained at a stable level in the following days. The thymus coefficient of *Nrf2^−/−^* mice was higher than that of *Nrf2^+/+^* mice 2–5 days after abdominal irradiation (*p* < 0.01), suggesting that *Nrf2* knockout promoted the thymus immune function of mice exposed to abdominal irradiation (Figure 2B).

### 2.3. Nrf2 Knockout Mitigated Peripheral Blood Lymphocyte Count Decreases of Mice after Abdominal Irradiation

**Figure 3:**
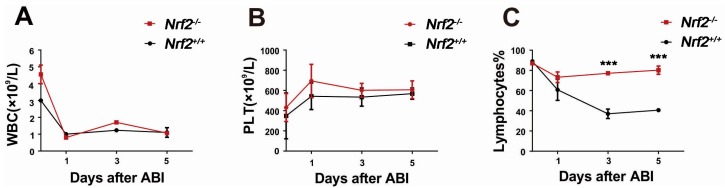
The effect of *Nrf2* knockout on various blood cells of mice after exposure to abdominal irradiation. Whole blood samples were obtained from the orbital sinus of *Nrf2^+/+^* mice and *Nrf2^−/−^* mice to count various cells at 1, 3, and 5 days post-abdominal irradiation. Then, (**A**) white blood cell (WBC), (**B**) blood platelets (PLT), and (**C**) lymphocytes were counted using a MEK-7222k hemocytometer. *** *p* < 0.001, *Nrf2^−/−^* group versus *Nrf2^+/+^* group.

To evaluate the blood system of *Nrf2^−/−^* mice and *Nrf2^+/+^* mice post-abdominal irradiation, the numbers of circulating white blood cell (WBC), blood platelets (PLT), and lymphocytes from the peripheral blood at 1, 3, and 5 days after abdominal irradiation were measured. The number of WBC decreased rapidly from the mice one day after abdominal irradiation and remained at a stable level in the following days. WBC counts showed no significant difference between *Nrf2^−/−^* mice and *Nrf2^+/+^* mice (Figure 3A), and there was no marked difference in PLT counts between *Nrf2^−/−^* mice and *Nrf2^+/+^* mice (Figure 3B). The lymphocyte count of *Nrf2^+/+^* mice decreased rapidly three days post-abdominal irradiation, whereas, the lymphocyte count of *Nrf2^−/−^* mice was little changed after abdominal irradiation (Figure 3C). The lymphocyte count of *Nrf2^−/−^* mice was higher than that of *Nrf2^+/+^* mice 3–5 days after abdominal irradiation (*p* < 0.001), suggesting that *Nrf2* knockout mitigated peripheral blood lymphocyte count decreases of mice following abdominal irradiation.

### 2.4. Nrf2 Knockout Attenuated DNA Damage to Peripheral Blood Lymphocytes of Mice After Abdominal Irradiation

**Figure 4:**
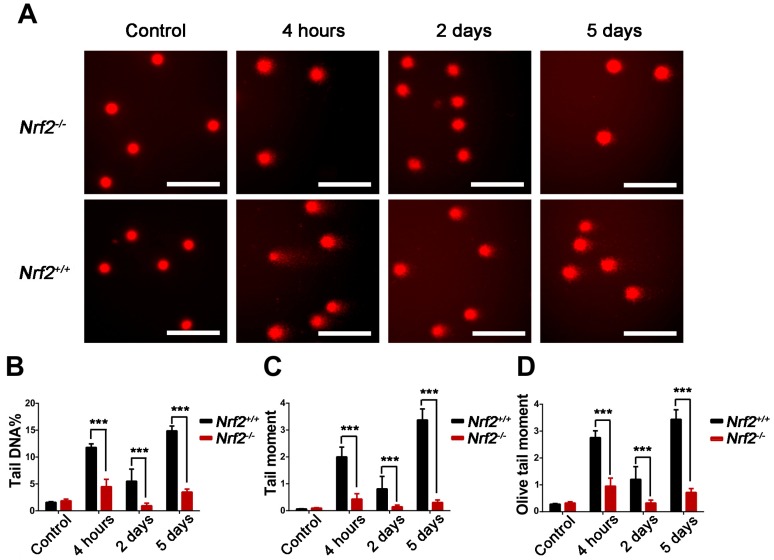
The effect of *Nrf2* knockout on DNA damage of mice after exposure to abdominal irradiation. Blood samples were obtained from the orbital sinus of *Nrf2^+/+^* mice and *Nrf2^−/−^* mice to isolate lymphocytes at 4 h, 2 days, and 5 days post-abdominal irradiation. Then, the alkali comet assay was adopted to determine DNA damage to lymphocytes from *Nrf2^+/+^* mice and *Nrf2^−/−^* mice. (**A**) Representative images of the DNA damage to peripheral blood lymphocytes. The comet tail length represents DNA damage. Scale bar, 50 μm; (**B**) graph displaying the DNA percentage in the comet tail (Tail DNA%) of lymphocytes; (**C**) graph showing the tail moment (TM) of lymphocytes; and (**D**) graph showing the olive tail moment (OTM) of lymphocytes. The bar graph represents mean ± SD obtained by analysing 200 cells/mouse (*n* = 4 mice/group). *** *p* < 0.001, *Nrf2^−/−^* group versus *Nrf2^+/+^* group.

We further evaluated the effect of Nrf2 on the DNA damage to peripheral blood lymphocytes from *Nrf2^−/−^* mice and *Nrf2^+/+^* mice by the comet assay. The DNA percentage in the comet tail (Tail DNA %), tail moment (TM), and olive tail moment (OTM) of lymphocytes represent the degree of DNA damage. As demonstrated in Figure 4, compared with those of *Nrf2^+/+^* mice, there was an obvious decrease in Tail DNA%, TM and OTM of *Nrf2^−/−^* mice at 4 h, 2 days, and 5 days after abdominal irradiation, respectively (*p* < 0.001). The results indicated that *Nrf2* knockout reduced abdominal irradiation-induced DNA damage to lymphocytes.

### 2.5. Nrf2 Knockout Alleviated the Damage to Crypt-Villus of the Small Intestine from Mice after Abdominal Irradiation

**Figure 5:**
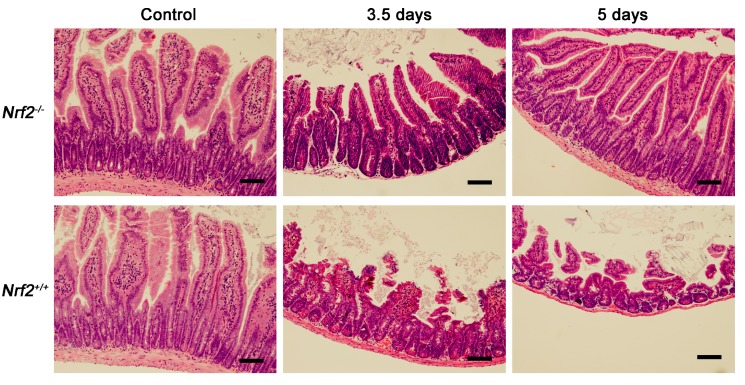
The effect of *Nrf2* knockout on the crypt-villus structure of the small intestine after exposure to abdominal irradiation. Representative haematoxylin and eosin (H and E)-stained sections of the small intestines. Small intestines from *Nrf2^+/+^* mice and *Nrf2^−/−^* mice were harvested for pathological examination after mice were euthanized 3.5 and 5 days post-abdominal irradiation. Scale bar, 100 μm.

To determine the effect of *Nrf2* knockout on radiation-induced intestinal injuries, the crypt-villus structure in the small intestine of mice was examined at 3.5 and five days post-abdominal irradiation by haematoxylin and eosin (H and E) staining. As shown in Figure 5, compared with that of the control mice, the crypt-villus structure in the small intestines of *Nrf2^−/−^* mice was well preserved at 3.5 and 5 days after abdominal irradiation. In contrast, the intestinal villi in *Nrf2^+/+^* mice were obviously stunted and blunt at 3.5 days after abdominal irradiation, and the number of intestinal crypts continued to decline at five days after abdominal irradiation. The results suggested that *Nrf2* knockout reduced abdominal irradiation-induced damage to the crypt-villus of the small intestine.

### 2.6. Nrf2 Knockout Decreased the Rate of Apoptosis of the Small Intestine from Mice after Abdominal Irradiation

**Figure 6:**
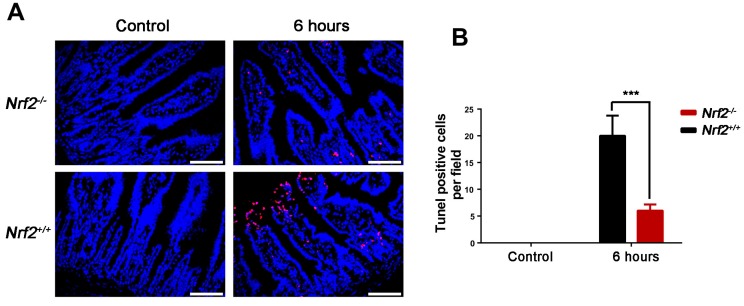
The effect of *Nrf2* knockout on the apoptosis of the small intestines after exposure to abdominal irradiation. The small intestines of *Nrf2^+/+^* mice and *Nrf2^−/−^* mice were harvested at 6 h post-abdominal irradiation for TUNEL assay. (**A**) Representative TUNEL staining images of the small intestines. Scale bar, 50 μm; (**B**) TUNEL-positive cells in a single field of view were quantified. The data were shown as mean ± SD. *** *p* < 0.001, *Nrf2^−/−^* group versus *Nrf2^+/+^* group.

IR induces apoptosis of intestinal epithelial cells, which is p53 dependent [18]. In the study, the rate of apoptosis of the small intestine was tested at 6 h after abdominal irradiation by terminal deoxynucleotidyl transferase dUTP nick end labeling (TUNEL) assay. As shown in Figure 6, the substantial numbers of apoptotic cells occurred in the intestinal villi of *Nrf2^+/+^* mice exposed to abdominal irradiation. Compared with that of *Nrf2^+/+^* mice, the rate of apoptosis of the small intestine from *Nrf2^−/−^* mice were significantly decreased (*p* < 0.001).

### 2.7. Nrf2 Knockout Resulted in an Increase in Lgr5^+^ Intestinal Stem Cells and Their Progeny of the Small Intestine from Mice after Abdominal Irradiation

**Figure 7:**
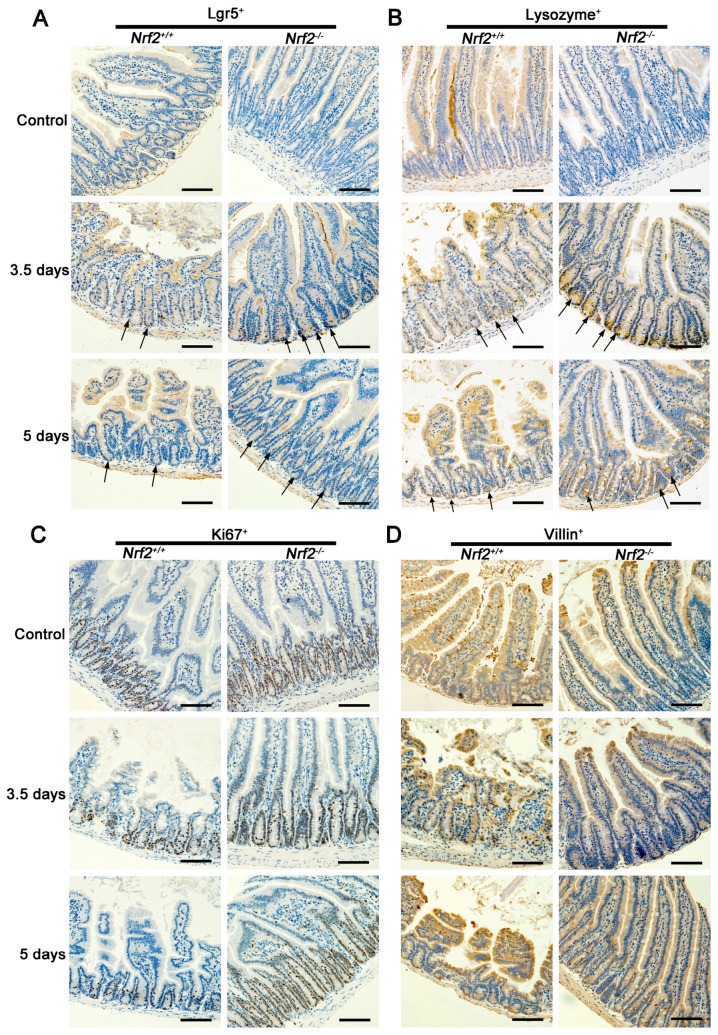
The effect of *Nrf2* knockout on the proliferation and differentiation ability of Lgr5^+^ of small intestine after exposure to abdominal irradiation. Small intestines of *Nrf2^+/+^* mice and *Nrf2^−/−^* mice were harvested at 3.5 and 5 days post-abdominal irradiation for immunohistochemical analysis. Representative immunohistochemical images for (**A**) Lgr5-, (**B**) Ki67-, (**C**) lysozyme-, and (**D**) Villin-stained sections of the small intestine. Scale bar, 100 μm.

*Lgr5^+^* intestinal stem cells are indispensable for intestinal regeneration following radiation [19]. To investigate the proliferation and differentiation ability of *Lgr5^+^* intestinal stem cells, the Lgr5^+^-, Ki67^+^-, Vil1in^+^-, and lysozyme^+^-stained sections, representing *Lgr5^+^* intestinal stem cells, transiently-amplifying cells, villus cells, and Paneth cells respectively, were observed at 3.5 and five days after abdominal irradiation. As shown in Figure 7A, the number of *Lgr5^+^* intestinal stem cells in *Nrf2^−/−^* mice at 3.5 and five days post abdominal irradiation was significantly higher than that of *Nrf2^+/+^* mice. Similarly, the numbers of Ki67^+^, villin^+^, and lysozyme^+^ cells of *Nrf2^−/−^* mice were also markedly higher than that of *Nrf2^+/+^* mice at 3.5 and five days post abdominal irradiation (Figure 7B–D). The data indicated that *Nrf2* knockout accelerated the regenerative response of radiation-induced intestinal injuries by promoting the proliferation and differentiation of *Lgr5^+^* intestinal stem cells in the small intestine.

### 2.8. Nrf2 Knockout Enhanced the Activation of NF-κB of the Small Intestine from Mice after Abdominal Irradiation

**Figure 8:**
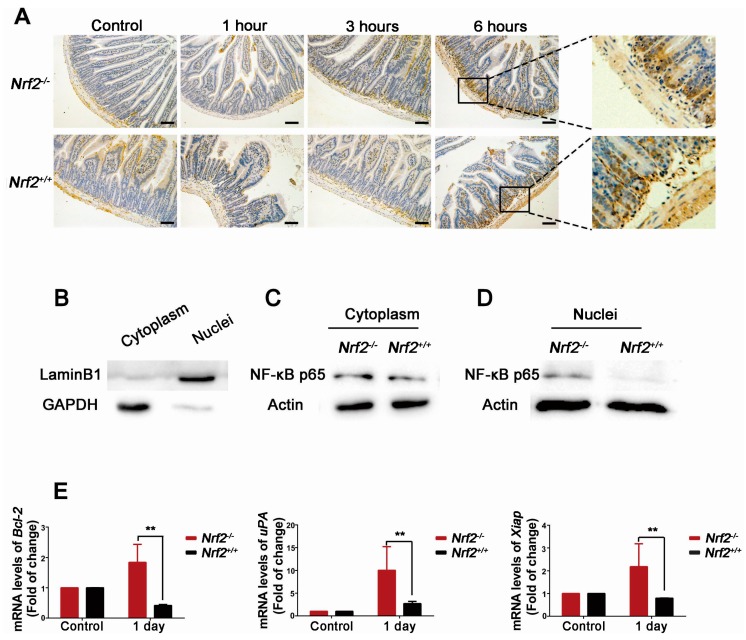
The effect of *Nrf2* knockout on activation of NF-κB of the small intestine after exposure to abdominal irradiation. (**A**) Immunohistochemical analysis for NF-κB p65-stained sections of the small intestine. Small intestines of *Nrf2^+/+^* mice and *Nrf2^−/−^* mice were harvested at 1, 3, and 6 h post-abdominal irradiation for immunohistochemical analysis. Scale bar, 100 μm; (**B**–**D**) The expression of NF-κB p65 in the cytoplasmic and nuclear lysates. Small intestines of mice were harvested at 6 h post-abdominal irradiation. The crypt cells were isolated from the small intestines to extract the cytoplasmic and nuclear lysates respectively. The cytoplasmic protein was identified by an anti-GAPDH antibody, and the nuclear protein was identified by an anti-LaminB1 antibody; (**E**) The mRNA levels of NF-κB target genes *Bcl-2*, *uPA*, and *Xiap*. Small intestines of mice were harvested at one day post-abdominal irradiation and total RNA was extracted to performed RT-PCR reaction. The data were expressed as the mean ± SD. ** *p* < 0.01, *Nrf2^−/−^* group versus the *Nrf2^+/+^* group.

To explore nuclear factor-κB (NF-κB) role in the protection effect of *Nrf2* knockout on radiation-induced intestinal injuries, the expression of NF-κB p65 of the small intestine at 1 h, 3 h, and 6 h after abdominal irradiation was examined by immunohistochemistry analysis. As demonstrated in Figure 8A, NF-κB p65 was accumulated in the nuclei of the intestinal crypt base. Compared with that of *Nrf2^+/+^* mice, the accumulation of NF-κB p65 of *Nrf2^−/−^* mice occurred at 3 h after abdominal irradiation, and was increased at 6 h after abdominal irradiation.

When it is activated under stress situations, NF-κB p65 immigrates from the cytoplasm into the nuclei of cells. The location of NF-κB p65 expression in the intestinal crypts cells at 6 h post-abdominal irradiation was verified by Western blot analysis. As shown in Figure 8D, the level of NF-κB p65 expression in the nuclei of the crypts cells from *Nrf2^−/−^* mice was higher than that of *Nrf2^+/+^* mice. In addition, the mRNA expression of NF-κB target genes *Bcl-2*, *uPA*, and *Xiap* in the small intestine after abdominal irradiation were examined. As shown in Figure 8E, the mRNA expression of *Bcl-2*, *uPA*, and *Xiap* in *Nrf2^−/−^* mice was significantly higher than that of *Nrf2^+/+^* mice at one day after abdominal irradiation (*p* < 0.01). The NF-κB target genes *Bcl-2*, *uPA*, and *Xiap* have the properties of anti-apoptosis and pro-survival. Taken together, these results indicated that the activation of NF-κB and upregulation of NF-κB target genes in *Nrf2^−/−^* mice may be involved in the event that *Nrf2* knockout alleviated radiation-induced intestinal injuries.

## 3. Discussion

Radiation-induced intestinal injuries, the side effects of abdominal or pelvic cavity tumour radiotherapy, severely affect the curative effect. In the study, *Nrf2^−/−^* mice following abdominal irradiation were used to investigate the effect of Nrf2 on radiation-induced intestinal injuries. It was reported that radiation-induced intestinal injuries occurred in a dose-dependent manner. Exposure of 12 Gy or lower-dose abdominal irradiation for the gastrointestinal tract was considered nonlethal to mice [20,21] and exposure of 14 Gy abdominal irradiation may be the maximum dose to allow for full intestinal recovery [22]. In our study, the dose of 13 Gy abdominal irradiation was adopted for the experimental mice. We found that *Nrf2* knockout promoted the survival rate and the body weight retention of mice after exposure to abdominal irradiation, indicating that *Nrf2* knockout had a protective effect on radiation-induced intestinal injuries.

Under physiological conditions, epithelial homeostasis is maintained by proliferative cells within crypts [23]. However, injuries to proliferative cells commonly occur in irradiated intestine. Various degrees of villus blunting and fusion may occur, with attenuation and hypertrophy of the villus epithelial cells [24] and severe loss of crypts [25], resulting in the destruction of epithelial homeostasis and epithelial integrity. An incomplete epithelium cannot easily maintain the absorptive and defensive functions of the intestine [26]. Thus, a rapid decline of body weight, and even death, were observed in the irradiated mice. Our findings demonstrated that the crypt-villus structure of the small intestines from *Nrf2^−/−^* mice was well preserved after abdominal irradiation and fewer apoptotic cells occurred in the small intestines, indicating that *Nrf2* knockout alleviated the degree of villus blunting and fusion and the loss of crypts, and preserved the epithelial integrity of mice post-abdominal irradiation. Consequently, it could explain why the survival rate and the body weight in *Nrf2^−/−^* mice after abdominal irradiation was increased. In addition, our results indicated that *Nrf2* knockout attenuated peripheral blood lymphocyte count decreases and DNA damage, as well as increased the thymus coefficient after abdominal irradiation. The boost in the blood system and the immune function may be the cause of the increase of the survival rate and the body weight in *Nrf2^−/−^* mice after abdominal irradiation.

In the present study, we found the radioprotective effect of *Nrf2* knockout on radiation-induced intestinal injuries. To explore the potential molecular mechanism, *Lgr5^+^* intestinal stem cells and their progeny in the small intestine were detected. The columnar cells of the intestinal crypt base expressing high levels of Wnt target gene *Lgr5* are defined as *Lgr5^+^* intestinal stem cells, which are indispensable for intestinal regeneration [19]. *Lgr5^+^* intestinal stem cells can divide into differentiated intestinal cell types, which migrate either upward into the villus to differentiate into functional cells, such as enterocytes, or downward into the crypt base to differentiate into transiently-amplifying cells [27,28]. Our findings demonstrated that the number of *Lgr5^+^* intestinal stem cells in *Nrf2^−/−^* mice after abdominal irradiation was increased and the increased *Lgr5^+^* intestinal stem cells differentiated into more transiently-amplifying cells, Paneth cells, and villus cells. Therefore, *Nrf2* knockout has the protective effect on radiation-induced intestinal injuries by promoting the proliferation and differentiation of *Lgr5^+^* intestinal stem cells.

NF-κB is a dimeric DNA binding protein, which is a member of the NF-κB/Rel family. It was reported that *Nrf2* absence was susceptibility to activation of NF-κB in vivo and in vitro [29,30,31,32,33], and NF-κB activation selectively protects the small intestine from radiation-induced intestinal injuries [34]. Consistently, the nuclear accumulation, the protein level of NF-κB p65 and NF-κB target genes in the intestinal crypt base of *Nrf2^−/−^* mice were significantly higher than that of *Nrf2^+/+^* mice. Our findings demonstrated that *Nrf2* knockout enhanced NF-κB activity of the intestinal crypt base in *Nrf2^−/−^* mice. The intestinal crypt base is the place that intestinal stem cells reside. As intestinal stem cells display high NF-κB activity [35] and NF-κB activation can make transformed cells grow and survive [36], it is plausible that NF-κB may promote the proliferation and differentiation of *Lgr5^+^* intestinal stem cells. Therefore, NF-κB activation in *Nrf2^−/−^* mice may be participated in the regeneration of radiation-induced intestinal injuries and may be one reason of the protective effect of *Nrf2* knockout on radiation-induced intestinal injuries.

However, it was reported that Nrf2 activation alleviated radiation-induced intestinal injuries of mice following total body irradiation [13,14,15,16]. We found that the effect of exposure to abdominal irradiation or total body irradiation on *Nrf2^−/−^* mice was completely opposite, that was, the survival of *Nrf2^−/−^* mice after abdominal irradiation was higher than that of *Nrf2^+^^/^^+^* mice, but the survival of *Nrf2^−/−^* mice after total body irradiation was lower than that of *Nrf2^+^^/^^+^* mice. It is known that abdominal irradiation can only lead to radiation-induced intestinal injuries, and total body irradiation can result in not only radiation-induced intestinal injuries, but also hematopoietic system injury. We speculate that the reason of the protective effect of *Nrf2* knockout on radiation-induced intestinal injuries may be attributed to different methods of radiation exposure. It has been reported that the roles and functions of Nrf2 in intestinal stem cells and hematopoietic stem cells (HSCs) are diverse. Hochmuth et al., found that CncC, which is a *Drosophila* homolog of Nrf2, was constitutively active in intestinal stem cells of *Drosophila* and maintained intestinal stem cells quiescent with low radical oxygen species concentration [37]. However, *Nrf2* deficiency attenuated the reconstitution capacity of hematopoietic stem cells after the transplantation, indicating that Nrf2 was required for the hematopoietic stem cells’ maintenance [6,38]. The different role of Nrf2 in intestinal stem cells and hematopoietic stem cells may explain why *Nrf2* knockout has the protective effect on radiation-induced intestinal injuries in this study, while Nrf2 alleviates radiation-induced hematopoietic system damage. It is important to note that non-*Nrf2* knockout mice were adopted to investigate the role of Nrf2 in radiation-induced intestinal injuries in previous studies, and *Nrf2* knockout mice were used to explore the effect of *Nrf2* knockout in the present study. The effect of *Nrf2* knockout mice on radiation-induced intestinal injuries was markedly different from that of non-*Nrf2* knockout mice, which may be one of reasons that our results were inconsistent with that of previous reports. However, it is necessary to further verify whether *Nrf2* knockout truly has the protective effect on radiation-induced intestinal injuries following abdominal irradiation, but not total body irradiation. In addition, the associated mechanisms by which *Nrf2* knockout facilitated the protective effect on radiation-induced intestinal injuries have yet to be elucidated thoroughly. Further study is needed to understand the contribution of radical oxygen species to radiation-induced intestinal injuries and explore how *Nrf2* knockout enhances the NF-κB activity.

## 4. Materials and Methods

### 4.1. Mice

Wild-type (Nrf2^+/+^) C57BL/6J mice were purchased from Beijing HFK Bioscience Co. Ltd (Beijing, China). Nrf2 knockout (Nrf2^−/−^) C57BL/6J mice were generously provided by Thomas W. Kensler of the University of Pittsburgh and backcrossed into the C57BL/6J background in our laboratory. All mice were housed in a temperature-controlled, specific pathogen free environment with a 12-h light/dark cycle, and fed standard chow and water. All experimental procedures and protocols were approved by the Animal Care and Ethics Committee of the Institute of Radiation Medicine (approval number: 170512, 5 August 2016), and performed according to the principles of the Institutional Animal Care and Ethics Committee guidelines.

### 4.2. Irradiation of Mice

Mice were anaesthetized with an intraperitoneal injection of 3.5% chloral hydrate (0.1 mL/10 g body weight) and placed on a platform to receive 13 Gy abdominal irradiation. The irradiated field was from the xiphoid process to the pubic symphysis. Mice were exposed to ionizing irradiation in a ^137^Cesium γ-ray irradiator (Atomic Energy of Canada, Chalk River, ON, Canada) at a rate of 1 Gy/min.

### 4.3. Analysis of the 30-Day Survival Rate

Mice were monitored for up to 30 days after 13 Gy abdominal irradiation or 7.2 Gy total body irradiation. The survival curves and the body weight curves were plotted by the Kaplan–Meier method and the ratio of body weight to initial weight, respectively.

### 4.4. Organ Coefficient Calculation

The spleen and thymus were collected and weighted after mice were euthanized. The organ coefficients were calculated by dividing individual organ weight (mg) with their body weight (g). The spleen coefficient = spleen weight (mg)/mice weight (g), the thymus coefficient = thymus weight (mg)/mice weight (g).

### 4.5. Peripheral Blood Cells Counts

Whole blood samples were obtained from the orbital sinus of mice. The numbers of various blood cell types, white blood cells (WBC), platelets (PLT), and lymphocytes were counted using a MEK-7222k hemocytometer (Nihonkohden Corp, Tokyo, Japan).

### 4.6. Analysis of DNA Damage to Peripheral Blood Lymphocytes

The comet assay, also called single-cell gel electrophoresis (SCGE), was performed under alkaline conditions as described by Banath et al. [39]. Whole blood was obtained from the orbital sinus of mice. Lymphocytes were harvested from peripheral blood using lymphocyte separation medium, mixed with low-melting agarose and fixed onto frosted slides pre-coated with normal melting agarose. The slides were immersed in cold, freshly made alkalinelysis solution at 4 °C for 2.5 h and then rinsed with PBS. Slides were placed in a horizontal electrophoresis device pre-filled with cold alkaline electrophoresis buffer for 20 min at 4 °C, followed by electrophoresis for 20 min (30 V, 300 mA). The slides were fixed in neutralization buffer, stained with ethidiumbromide (2.5 μg/mL), and visualized by a ECLIPSE 90i fluorescence microscopy (Nikon, Tokyo, Japan). Two hundred cells were analysed using the Comet Assay Software Project (CASP, Wroclaw University, Poland) [40], and the DNA percentage in the comet tail (Tail DNA%), tail moment (TM), and olive tail moment (OTM) were recorded to describe DNA damage to the lymphocytes.

### 4.7. Pathological Analysis

Dissected small intestines were harvested after mice were euthanized and fixed in neutral formalin for 24 h prior to being embedded in paraffin. Paraffin-embedded small intestines were cut into 5 μm sections at the maximum cross-section. Sections were stained with haematoxylin/eosin and alcian blue.

### 4.8. Immunohistochemical Analysis

Paraffin-embedded sections were dewaxed and rehydrated. The sections were then immersed in Tris/EDTA (pH 9.0) and incubated for 15 min at 98 °C for antigen retrieval. The sections were incubated with serum for 30 min at room temperature to block non-specific antigen-binding sites. Anti-Ki67 antibody, anti-NF-κB p65 antibody, anti-Lgr5 antibody (Abcam, Cambridge, MA, USA), anti-lysozyme antibody, and anti-Villin antibody (Proteintech, Chicago, IL, USA) was diluted as recommended by the manufacturer, added to the sections, and incubated overnight at 4 °C. Unbound antibodies were washed away with PBS. Sections were incubated in 0.3% H_2_O_2_ for 15 min to block endogenous peroxidase. Secondary antibody was added and incubated at 37 °C for 2 h. DAB was added to detect positive cells.

### 4.9. Apoptosis Assay

The apoptosis assay was tested using one step TUNEL apoptosis assay kit (Beyotime, Shanghai, China). Paraffin-embedded sections were dewaxed and rehydrated. The sections were then treated with proteinase K for 5 min in a 37 °C water bath and incubated with TUNEL detection liquid for 1 h at 37 °C. After washes, sections were counterstained with DAPI. These slides were observed under an ECLIPSE 90i fluorescence microscope (Nikon). TUNEL-positive cells in a field of view were quantified.

### 4.10. Isolation of Crypt Single Cells

The method of isolating the intestinal crypts of mice was described by Mahe et al. [41]. Briefly, the small intestines of mice were sliced and opened longitudinally. After washing with ice-cold PBS, the small intestines were cut into small pieces and then incubated in ice-cold PBS containing 2 mM EDTA for 30 min. After rinsing twice with ice-cold PBS, the fragments were resuspended in ice-cold PBS containing 54.9 mM d-sorbitol and 43.4 mM sucrose, and filtered with a 70-mm filter. After centrifuging, the pellet was incubated in pancreatin until it was digested to single cells, and filtered with a 40 μm cell strainer.

### 4.11. Preparation of Cytoplasmic and Nuclear Extracts

Cytoplasmic and nuclear extracts were prepared from crypts single cells using NE-PER™ nuclear and cytoplasmic extraction reagents (ThermoFisher, Rockford, IL, USA) according to the manufacturer’s instructions. The single cells pellet was suspended with ice-cold cytoplasmic extraction reagent I for 10 min and then added ice-cold cytoplasmic extraction reagent II. After centrifuging, the supernatant (cytoplasmic extract) was collected. The insoluble fraction was suspended with nuclear extraction reagent. The supernatant (nuclear extract) was collected following centrifuging.

### 4.12. Western Blot Analysis

The concentrations of protein lysates were quantified using a BCA protein kit (Beyotime). Samples containing equal amounts of protein (20 μg) were mixed with loading buffer containing 5% 2-mercaptoethanol, heated for 10 min at 99 °C, and loaded onto a 10% SDS-PAGE gel. After electrophoresing, the proteins from the gel were then transferred onto polyvinylidene difluoride membranes. These membranes were blocked with 5% milk and 0.1% Tween 20 in Tris-buffered saline, and incubated overnight at 4 °C with anti-NF-κB p65 (Abcam), anti-LaminB1, anti-GAPDH and anti-β-Actin (Proteintech). Then, the appropriate horseradish peroxide-conjugated secondary antibody was added on the membranes at room temperature. Finally, the proteins were detected with chemiluminescent substrate.

### 4.13. RNA Isolation and Real-Time Polymerase Chain Reaction

Total RNA was extracted from the small intestines using TRIzol Reagent according to the manufacturer’s instructions (Invitrogen, Carlsbad, CA, USA). A reverse transcription reaction was performed with 10 μg aliquot of each RNA sample using oligo-dT random primers and reverse transcriptase (Takara, Dalian, China), and real-time PCR was performed using a SYBR Premix Ex TaqTM II (Takara). Primer sequences were as follows:

Bcl-2: 5′-AGGATTGTGGCCTTCTTTGA-3′ (forward) and 5′-CAGATGCCGGTTCAGGTACT-3′ (reverse); XIAP: 5′-TGGAGACTCAGCTGTTGGAA-3′ (forward) and 5′-CTGGCCATTTTGGATACCAG-3′ (reverse); uPA: 5′-CGCACACTGCTTCATTCAAC-3′ (forward) and 5′-GCTGCTCCACCTCAAACTTC-3′ (reverse).

### 4.14. Statistical Analysis

The data were analysed using Student’s *t*-test and expressed as the mean ± SD. The Kaplan–Meier method was used to analyse animal survival curves. A *p*-value of <0.05 was taken to be significant.

## References

[1] Potten C.S. (2004). Radiation, the ideal cytotoxic agent for studying the cell biology of tissues such as the small intestine. Radiat. Res..

[2] Moi P., Chan K., Asunis I., Cao A., Kan Y.W. (1994). Isolation of NF-E2-related factor 2 (Nrf2), a NF-E2-like basic leucine zipper transcriptional activator that binds to the tandem NF-E2/AP1 repeat of the β-globin locus control region. Proc. Natl. Acad. Sci. USA..

[3] Hirotsu Y., Katsuoka F., Funayama R., Nagashima T., Nishida Y., Nakayama K., Engel J.D., Yamamoto M. (2012). Nrf2-MafG heterodimers contribute globally to antioxidant and metabolic networks. Nucleic Acids Res..

[4] Hayes J.D., Dinkova-Kostova A.T. (2014). The Nrf2 regulatory network provides an interface between redox and intermediary metabolism. Trends Biochem. Sci..

[5] Rwigema J.C., Beck B., Wang W., Doemling A., Epperly M.W., Shields D., Goff J.P., Franicola D., Dixon T., Frantz M.C. (2011). Two strategies for the development of mitochondrion-targeted small molecule radiation damage mitigators. Int. J. Radiat. Oncol. Biol. Phys..

[6] Merchant A.A., Singh A., Matsui W., Biswal S. (2011). The redox-sensitive transcription factor Nrf2 regulates murine hematopoietic stem cell survival independently of ROS levels. Blood.

[7] Zhao Q., Mao A., Guo R., Zhang L., Yan J., Sun C., Tang J., Ye Y., Zhang Y., Zhang H. (2017). Suppression of radiation-induced migration of non-small cell lung cancer through inhibition of Nrf2-Notch axis. Oncotarget.

[8] Zhao Q., Mao A., Yan J., Sun C., Di C., Zhou X., Li H., Guo R., Zhang H. (2016). Downregulation of Nrf2 promotes radiation-induced apoptosis through Nrf2 mediated Notch signaling in non-small cell lung cancer cells. Int. J. Oncol..

[9] Jeong Y., Hoang N.T., Lovejoy A., Stehr H., Newman A.M., Gentles A.J., Kong W., Truong D., Martin S., Chaudhuri A. (2017). Role of KEAP1/NRF2 and TP53 mutations in lung squamous cell carcinoma development and radiation resistance. Cancer Discov..

[10] Kim J.H., Thimmulappa R.K., Kumar V., Cui W., Kumar S., Kombairaju P., Zhang H., Margolick J., Matsui W., Macvittie T. (2014). NRF2-mediated Notch pathway activation enhances hematopoietic reconstitution following myelosuppressive radiation. J. Clin. Investig..

[11] Chute J.P. (2014). NRF2 mitigates radiation-induced hematopoietic death. J. Clin. Investig..

[12] Rana T., Schultz M.A., Freeman M.L., Biswas S. (2012). Loss of Nrf2 accelerates ionizing radiation-induced bone loss by upregulating RANKL. Free Radic. Biol. Med..

[13] Kim S.B., Pandita R.K., Eskiocak U., Ly P., Kaisani A., Kumar R., Cornelius C., Wright W.E., Pandita T.K., Shay J.W. (2012). Targeting of Nrf2 induces DNA damage signaling and protects colonic epithelial cells from ionizing radiation. Proc. Natl. Acad. Sci. USA..

[14] Kim S.B., Ly P., Kaisani A., Zhang L., Wright W.E., Shay J.W. (2013). Mitigation of radiation-induced damage by targeting EGFR in noncancerous human epithelial cells. Radiat. Res..

[15] Dutta A., Gupta M.L., Kalita B. (2015). The combination of the active principles of *Podophyllum hexandrum* supports early recovery of the gastrointestinal system via activation of Nrf2–HO-1 signaling and the hematopoietic system, leading to effective whole-body survival in lethally irradiated mice. Free Radical Res..

[16] Wang K.P., Zhang C., Zhang S.G., Liu E.D., Dong L., Kong X.Z., Cao P., Hu C.P., Zhao K., Zhan Y.Q. (2015). 3-(3-pyridylmethylidene)-2-indolinone reduces the severity of colonic injury in a murine model of experimental colitis. Oxid. Med. Cell Longev..

[17] Berhane H., Epperly M.W., Cao S., Goff J.P., Franicola D., Wang H., Greenberger J.S. (2013). Radioresistance of bone marrow stromal and hematopoietic progenitor cell lines derived from Nrf2^−/−^ homozygous deletion recombinant-negative mice. In Vivo.

[18] Kirsch D.G., Santiago P.M., di Tomaso E., Sullivan J.M., Hou W.S., Dayton T., Jeffords L.B., Sodha P., Mercer K.L., Cohen R. (2010). p53 Controls radiation-induced gastrointestinal syndrome in mice independent of apoptosis. Science.

[19] Metcalfe C., Kljavin N.M., Ybarra R., de Sauvage F.J. (2014). Lgr5^+^ stem cells are indispensable for radiation-induced intestinal regeneration. Cell Stem Cell.

[20] Bhanja P., Saha S., Kabarriti R., Liu L., Roy-Chowdhury N., Roy-Chowdhury J., Sellers R.S., Alfieri A.A., Guha C. (2009). Protective role of R-spondin1, an intestinal stem cell growth factor, against radiation-induced gastrointestinal syndrome in mice. Plos ONE.

[21] Hua G., Thin T.H., Feldman R., Haimovitz-Friedman A., Clevers H., Fuks Z., Kolesnick R. (2012). Crypt base columnar stem cells in small intestines of mice are radioresistant. Gastroenterology.

[22] Gong W., Guo M., Han Z., Wang Y., Yang P., Xu C., Wang Q., Du L., Li Q., Zhao H. (2016). Mesenchymal stem cells stimulate intestinal stem cells to repair radiation-induced intestinal injury. Cell Death Dis..

[23] Umar S. (2010). Intestinal stem cells. Curr. Gastroenterol. Rep..

[24] Potten C.S., Merritt A., Hickman J., Hall P., Faranda A. (1994). Characterization of radiation-induced apoptosis in the small intestine and its biological implications. Int. J. Radiat. Biol..

[25] Merritt A.J., Potten C.S., Kemp C.J., Hickman J.A., Balmain A., Lane D.P., Hall P.A. (1994). The role of p53 in spontaneous and radiation-induced apoptosis in the gastrointestinal tract of normal and p53-deficient mice. Cancer Res..

[26] Ripp T., Ryabov V., Karpov R. (2007). Significance changes endothelial dysfunction after enhanced external conterpulsation treatment in hypertensive patients with stable angina. J. Hypertens..

[27] Sato T., Clevers H. (2013). Growing self-organizing mini-guts from a single intestinal stem cell: Mechanism and applications. Science.

[28] Sato T., Vries R.G., Snippert H.J., van de Wetering M., Barker N., Stange D.E., van Es J.H., Abo A., Kujala P., Peters P.J. (2009). Single Lgr5 stem cells build crypt-villus structures in vitro without a mesenchymal niche. Nature.

[29] Jin W., Wang H., Yan W., Xu L., Wang X., Zhao X., Yang X., Chen G., Ji Y. (2008). Disruption of Nrf2 enhances upregulation of nuclear factor-κB activity, proinflammatory cytokines, and intercellular adhesion molecule-1 in the brain after traumatic brain injury. Mediat. Inflamm..

[30] Mao L., Wang H., Qiao L., Wang X. (2010). Disruption of Nrf2 enhances the upregulation of nuclear factor-κB activity, tumor necrosis factor-α, and matrix metalloproteinase-9 after spinal cord injury in mice. Mediat. Inflamm..

[31] Pan H., Wang H., Wang X., Zhu L., Mao L. (2012). The absence of Nrf2 enhances NF-κB-dependent inflammation following scratch injury in mouse primary cultured astrocytes. Mediat. Inflamm..

[32] Cuadrado A., Martín-Moldes Z., Ye J., Lastres-Becker I. (2014). Transcription factors NRF2 and NF-κB are coordinated effectors of the Rho family, GTP-binding protein RAC1 during inflammation. J. Biol. Chem..

[33] Jin W., Zhu L., Guan Q., Chen G., Wang Q.F., Yin H.X., Hang C.H., Shi J.X., Wang H.D. (2008). Influence of Nrf2 genotype on pulmonary NF-κB activity and inflammatory response after traumatic brain injury. Ann. Clin. Lab. Sci..

[34] Wang Y., Meng A., Lang H., Brown S.A., Konopa J.L., Kindy M.S., Schmiedt R.A., Thompson J.S., Zhou D. (2004). Activation of nuclear factor κB in vivo selectively protects the murine small intestine against ionizing radiation-induced damage. Cancer Res..

[35] Van der Heijden M., Zimberlin C.D., Nicholson A.M., Colak S., Kemp R., Meijer S.L., Medema J.P., Greten F.R., Jansen M., Winton D.J. (2016). Bcl-2 is a critical mediator of intestinal transformation. Nat. Commun..

[36] Aggarwal B.B. (2000). Apoptosis and nuclear factor-κB: A tale of association and dissociation. Biochem. Pharmacol..

[37] Hochmuth C.E., Biteau D., Bohmann D., Jasper H. (2011). Redox regulation by Keap1 and Nrf2 controls intestinal stem cell proliferation in *Drosophila*. Cell Stem Cell.

[38] Tsai J.J., Dudakov J.A., Takahashi K., Shieh J.H., Velardi E., Holland A.M., Singer N.V., West M.L., Smith O.M., Young L.F. (2013). Nrf2 regulates haematopoietic stem cell function. Nat. Cell Biol..

[39] Banath J.P., Fushiki M., Olive P.L. (1998). Rejoining of DNA single- and double-strand breaks in human white blood cells exposed to ionizing radiation. Int. J. Radiat. Biol..

[40] Konca K., Lankoff A., Banasik A., Lisowska H., Kuszewski T., Gozdz S., Koza Z., Wojcik A. (2003). A cross-platform public domain PC image-analysis program for the comet assay. Mutat. Res..

[41] Mahe M.M., Aihara E., Schumacher M.A., Zavros Y., Montrose M.H., Helmrath M.A., Sato T., Shroyer N.F. (2013). Establishment of gastrointestinal epithelial organoids. Curr. Protoc. Mouse Biol..

